# Functional Outcomes and Self-Reported Quality of Life in Patients with Facial Nerve Impairment Following Vestibular Schwannoma Surgery

**DOI:** 10.3390/diagnostics14212387

**Published:** 2024-10-26

**Authors:** Leonardo Franz, Silvia Montino, Anna Agostinelli, Giulia Tealdo, Diego Cazzador, Elisabetta Zanoletti, Gino Marioni

**Affiliations:** 1 Phoniatrics and Audiology Unit, Department of Neuroscience DNS, Padova University, 31100 Treviso, Italy; leonardo.franz@unipd.it; 2 Section of Otolaryngology, Department of Neuroscience DNS, Padova University, 35100 Padova, Italy; silvia.montino@unipd.it (S.M.); anna.agostinelli@unipd.it (A.A.); giulia.tealdo@aopd.veneto.it (G.T.); diego.cazzador@unipd.it (D.C.); elisabetta.zanoletti@unipd.it (E.Z.)

**Keywords:** facial nerve palsy, vestibular schwannoma, quality of life, synkinesis, patient-reported outcomes

## Abstract

Objective: The aim of this observational retrospective study was to report quality of life (QoL) in patients with postoperative facial nerve (FN) palsy after vestibular schwannoma (VS) surgery, investigating clinical factors related to functional outcomes. Methods: Forty-eight consecutive patients (M:F 25:23; median age: 52.5 years) with facial palsy following surgery for sporadic VS were considered retrospectively. FN palsy was graded by using the Sunnybrook facial grading system (SBFGS), while postoperative QoL and subjective functional aspects were assessed by using the Penn Acoustic Neuroma Quality of Life (PANQOL) Scale, the Synkinesis Assessment Questionnaire, and questions on eating and drinking. Results: A significant correlation emerged between all Sunnybrook scores and median PANQOL domain regarding facial function. Increasing overall SBFGS scores were associated with reduced risk of slow chewing on the affected side (*p* = 0.004), lack of masticatory strength (*p* = 0.025), masticatory fatigue (*p* < 0.001), accumulation of food in the oral vestibule (*p* < 0.001), difficulty in drinking from a glass (*p* = 0.019), and fluid spillage while drinking (*p* = 0.016). Conclusions: This study suggests that the clinical evaluation of patients with FN palsy after VS surgery should be integrated with patient reports about functional outcomes and perceived QoL to help clinicians guide rehabilitation choices.

## 1. Introduction

Vestibular schwannoma (VS) is a benign tumor originating from Schwann cells. It involves unpredictable hearing loss over time, vestibular symptoms, less frequently facial nerve impairment, and in large tumors, neurological signs due to cerebellar–brainstem compression. Surgery is currently providing excellent outcomes in small tumors [1,2], in terms of related morbidity, cure of disease, and facial nerve preservation. The issue of hearing preservation in small tumors is more complex and strongly influenced by the preoperative hearing status. It is known that VS size negatively impacts surgical facial nerve preservation rates, as confirmed by better outcomes reported in small tumors [1] than in medium- [2] and large-sized ones [3,4]. In small VSs, early surgery may represent a viable option in the case of a growing tumor, to maximize the goal of definitive cure with the best chances of facial nerve preservation [1,2,5,6]. Surgical timing is also dictated by hearing status, tumor size, and patient’s conditions. Regardless of the type of approach, VS removal may lead to peripheral facial paralysis [7,8] with an increasing risk in relation to tumor size. Peripheral facial nerve paralysis potentially results in permanent functional and aesthetic sequelae [9]. This condition significantly impacts patients’ quality of life by impairing face aesthetics, resulting in facial asymmetry both at rest and during muscle contraction, as well as mimic non-verbal communication function [10,11]. Swallowing disorders in the oral phase have also been reported, with difficulties in bolus and saliva control due to the reduced lips’ muscles’ strength [12], mainly in the lower one [13]. This also reflects on masticatory function, leading to a difficulty in chewing on the injured side and to less efficient bolus formation [14,15] and on speech, resulting in articulation errors, more specifically of bi-labial consonants and labio-dental fricative [13].

The functional and aesthetic impairment related to facial nerve paralysis is often perceived by patients as a major disability, causing a significant decrease in quality of life, with detrimental psycho-social effects, including social withdrawal and depression [16]. In clinical practice, the main tools commonly used to score the severity of facial nerve palsy are the House–Brackmann (HB) scale [17] and the Sunnybrook facial grading system [18]. However, such clinometric tools cannot provide information on how patients perceive facial nerve dysfunctions and how much this deficit impacts their quality of life. This is even more relevant when the facial dysfunction is associated with hearing loss and vestibular impairment [19], which are not uncommon sequelae of VS surgery. More recently, some clinimetric tools in the form of questionnaires to assess quality of life in VS patients have been proposed and validated [20].

The main aim of this study was to report on patients’ perceived quality of life in a group of patients who experienced a certain degree of facial paresis after VS surgery and for whom appointments were then scheduled in the rehabilitative setting over different periods of follow-up. A secondary aim was to investigate which factors had a major impact on the clinical outcome and self-assessed perception.

## 2. Materials and Methods

### 2.1. Setting

This study was conducted in accordance with the Declaration of Helsinki. Data were examined in compliance with Italian privacy and sensitive data laws and with the in-house rules of our institution. All patients gave their written consent for the medical procedures and signed a disclosure form on privacy in managing their clinical data for scientific purposes.

Data were collected and reported according to the STROBE statement (https://www.strobe-statement.org/checklists, accessed on 7 March 2022).

Data were retrospectively retrieved over a six-month period (March to October 2022) in the outpatient setting of the speech therapy, swallowing, and facial nerve rehabilitation team. All patients had been operated on for VS in our Institution (Otolaryngology Section, Lateral Skull Base Unit, University Hospital of Padova (Italy)), by the same surgical équipe, between 2012 and 2022.

Every patient who is admitted to the VS surgery program at our institution is planned to be evaluated in the setting of a targeted rehabilitation program according to the patient’s clinical situation. In the first postoperative year, follow-up is scheduled in the early postoperative days, at discharge, and then at 1, 3, 6, and 12 months postoperatively. Subsequently, it varies according to the patients’ needs, difficulties, and improvements and is targeted to each condition. The six-month period evaluation for the present study involved patients admitted accordingly and was followed by different lengths of follow-up.

### 2.2. Patients

This retrospective investigation involved a group of patients who had undergone surgery for sporadic VS at different times and for whom, due to various degrees of postoperative facial palsy, as assessed at one month postoperatively, appointments were scheduled in the outpatient rehabilitative setting of our institution. Only the cases with a minimum follow-up of 6 months were analyzed. Each patient had been operated on by the same surgical team via either trans-labyrinthine, retro-sigmoid, retro-labyrinthine/pre-sigmoid, middle fossa, or trans-otic approaches. 

The inclusion criteria were the following:

1. Age over 18 years;
2. Diagnosis of sporadic VS;
3. Postoperative onset of HB > 2 facial nerve paralysis, assessed on the 30th POD;
4. Presence of anatomically preserved FN or, in case of intraoperative gross anatomical damage, direct nerve reconstruction by a cable graft;
5. Minimum follow-up after surgery of six months.

Patients with diagnosis of neurofibromatosis type 2 (NF2)-related VS were excluded. 

### 2.3. Clinical Evaluation of Facial Palsy and Quality of Life

Clinical data including age at evaluation, sex, diagnosis, and tumor dimension were collected. Data regarding the procedure were obtained from surgical records. For each patient, facial nerve palsy was clinically evaluated at the last available follow-up and graded by a trained speech therapist and an otolaryngologist, using the Italian version of the Sunnybrook facial grading system (SBFGS) [21]. Data on both overall and partial scores (resting symmetry, symmetry of voluntary movement, and synkinesis) were collected.

To characterize and quantify postoperative quality of life, at the last available follow-up, each patient completed the Penn Acoustic Neuroma Quality of Life (PANQOL) Scale [20,22], the Synkinesis Assessment Questionnaire (SAQ) [23], and a questionnaire related to the difficulties met by patients with facial palsy in eating and drinking [24]. Both the PANQOL Scale and the SAQ required the patient to score each item on a 1-to-5 scale according to the degree of agreement with each statement and according to the frequency of synkinetic movements, respectively. The PANQOL and SAQ global scores were then calculated and converted into a score from 0 to 100. For the PANQOL Scale, a score of 100 corresponded to the highest perception of the quality of life, whereas for the SAQ, the highest score described the presence of synkinesis. Furthermore, answers to the PANQOL Scale were analyzed according to different domains: Anxiety, Facial Dysfunction, General Health, Balance, Hearing loss, Energy, and Pain. Each domain has a 0 to 100 score, where 100 corresponds to the worst quality of life. The PANQOL total score is calculated as the average score of all domain scores [22].

The proportion of yes/no answers in the questionnaire about eating and drinking was also calculated.

### 2.4. Statistical Analysis

Continuous variables were summarized with medians and interquartile ranges (IQRs), while categorical ones were described in terms of counts and percentages in each category.

Fisher’s exact test and Mann–Whitney U test were applied as appropriate. Correlation between continuous variables was expressed in terms of Spearman’s rank correlation coefficient. Logistic regression was used to explore the association between Sunnybrook scores and the dichotomous variables about eating and drinking functions.

A between-groups analysis, based on the above-mentioned tests, as appropriate, was also performed to compare QoL outcomes in patients with intraoperative preservation of FN vs. those with direct graft reconstruction.

For all the employed tests, a *p*-value < 0.05 was considered indicative of statistical significance, while values between 0.05 and 0.10 were assumed to indicate a statistical trend towards significance.

The STATA 16.0 IC statistical package (StataCorp LP, College Station, TX, USA) was used for all analyses.

## 3. Results

### 3.1. Participants, Descriptive Data, and Overall Clinical Features

Forty-eight VS cases (23 females and 25 males) with postoperative facial nerve palsy met the inclusion criteria. Overall, the median age at surgery of these patients was 52.5 years [45.5–59 years]. The median tumor size was 15 mm [10–23 mm]. Koos grading was calculated as 23% for grade 1, 30% for grades 2 and 3 each, and 17% for grade 4.

Preoperative facial nerve function was normal in 46 out of 48 cases (1 case had House–Brackmann grade 2, and 1 had grade 3).

The surgical approach was trans-labyrinthine in 29 cases, retro-sigmoid in 16, retro-labyrinthic/pre-sigmoid in 1, middle fossa in 1, and trans-otic in 1. Facial nerve reconstruction with graft was performed in nine patients. Among these cases, Koos grade 2 was present in 11% (1/9), grade 3 in 55.6% (5/9), and grade 4 in 33.3% (3/9). No VS with Koos grade 1 was observed.

At the last available follow-up (median: 27.5 months [13.5–67 months]), no patient showed tumor recurrence.

### 3.2. Distribution of Facial Nerve Function Parameters and Self-Reported Quality of Life

**Figure 1:**
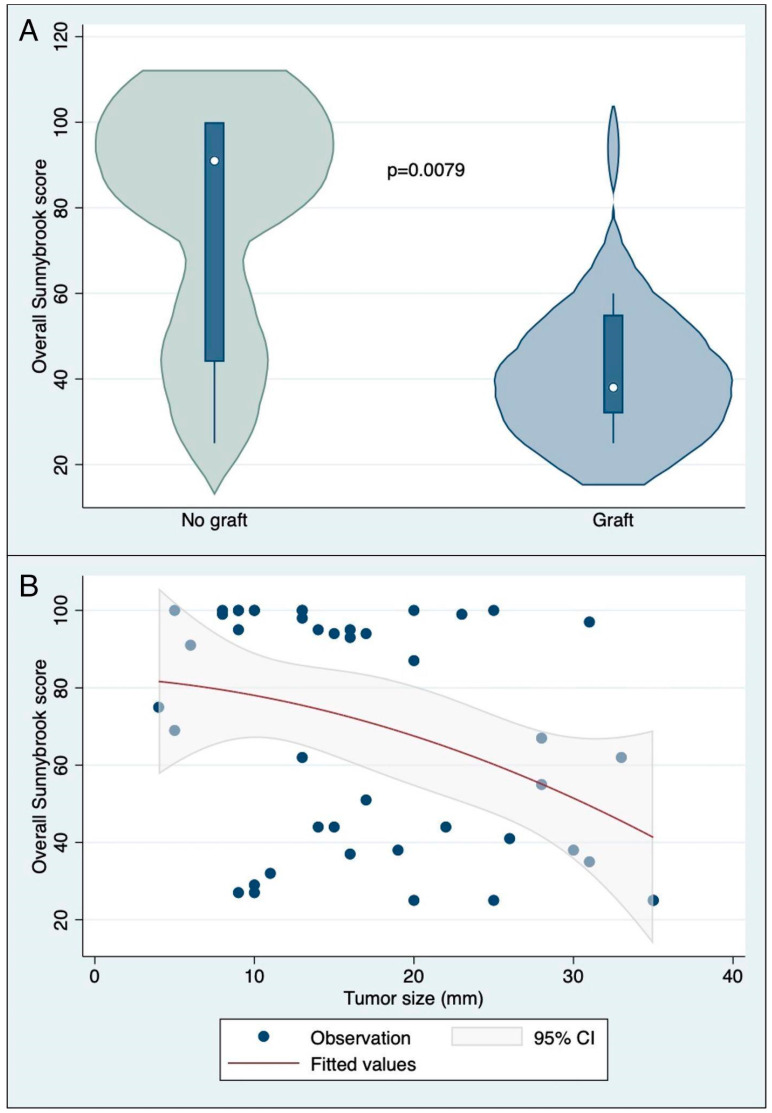
(**A**) Violin plot showing the distribution of overall Sunnybrook scores of patients who underwent facial nerve graft and those who did not; (**B**) correlation between tumor size and Sunnybrook score (Spearman’s rho: −0.3898; *p* = 0.0081).

At the last available follow-up, the median postoperative overall Sunnybrook score was 68 [39.5–98.5]. The overall Sunnybrook score was significantly lower in patients who underwent facial nerve graft compared with those who did not (Mann–Whitney U test, *p* = 0.0079; see also Figure 1A).

According to Koos classification, the median Sunnybrook scores were 99 (83–100), 51 (34.5–94), 60 (39.5–94.5), and 41 (32.5–63.2) for grades 1, 2, 3, and 4, respectively.

The distribution of the overall and partial scores across the different subgroups is summarized in Table 1. A significant (although weak) negative correlation was found between tumor size and Sunnybrook score (Spearman’s rho: −0.3898; *p* = 0.0081; see also Figure 1B).

The median overall PANQOL score was 74.62 [66.15–85.48]. No significant differences in terms of overall and partial PANQOL scores were found between patients who underwent facial nerve grafting and those who did not, except for the partial facial dysfunction score (Mann–Whitney U test, *p* = 0.0255; see also Table 1).

The median overall SAQ score was 22.22 [17.78–34.44] and did not vary significantly according to the presence of the graft (Mann–Whitney U test, *p* = 0.4918).

The distribution of dichotomous outcomes related to eating and drinking functions is summarized in Table 2.

### 3.3. Association Between Sunnybrook Scores and Self-Reported Quality of Life Outcomes

**Figure 2:**
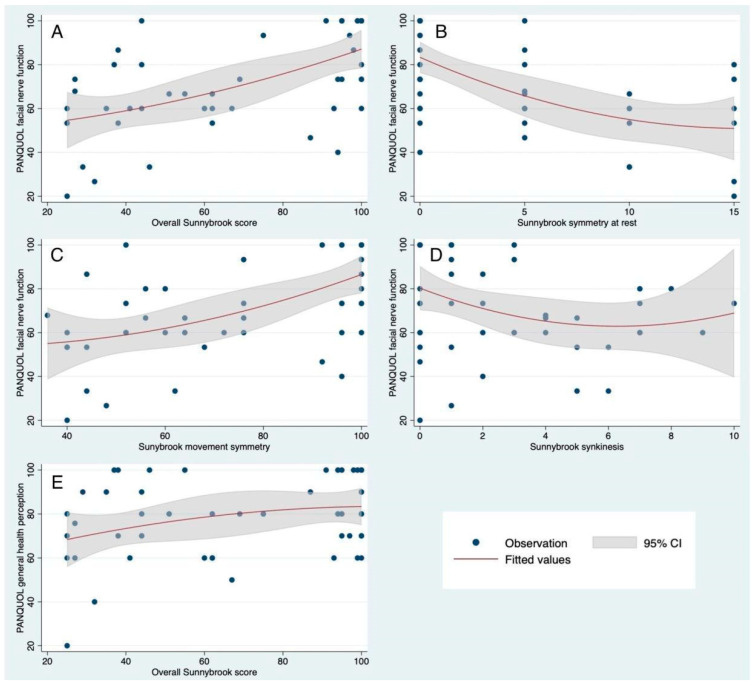
Correlation between all Sunnybrook scores and the specific PANQOL domain regarding facial function: (**A**) Sunnybrook total (Spearman’s rho: 0.6150, *p* < 0.001); (**B**) symmetry at rest score (Spearman’s rho: −0.5744, *p* < 0.001); (**C**) movement symmetry score (Spearman’s rho: 0.6014, *p* < 0.001); (**D**) synkinesis score (Spearman’s rho: −0.2899, *p* = 0. 0457). (**E**) correlation between overall Sunnybrook score and specific PANQOL domain regarding general health perception (Spearman’s rho: 0.2619, *p* = 0. 0721).

Although the overall and partial Sunnybrook scores did not correlate with the overall PANQOL score (*p* = 0.3227, *p* = 0.2677, *p* = 0.3049, and *p* = 0.6540 for Sunnybrook total, symmetry at rest, movement symmetry, and synkinesis, respectively), a significant correlation emerged between all Sunnybrook scores and the specific PANQOL domain regarding facial function (Spearman’s rho: 0.6150, *p* < 0.001 for Sunnybrook total; Spearman’s rho: −0.5744, *p* < 0.001 for symmetry at rest; Spearman’s rho: 0.6014, *p* < 0.001 for movement symmetry; Spearman’s rho: −0.2899, *p* = 0. 0457 for synkinesis; see also Figure 2A–D). A statistical trend toward correlation between overall Sunnybrook score and the specific PANQOL domain regarding general health perception was also found (Spearman’s rho: 0.2619, *p* = 0. 0721; Figure 2E).

### 3.4. Association Between Sunnybrook Scores and Self-Reported Facial Nerve Functional Outcomes

The overall Sunnybrook score and the partial one based on movement symmetry were negatively correlated with the SAQ score (Spearman’s rho: −0.5355, *p* = 0.0001 for Sunnybrook total; Spearman’s rho: −0.5269, *p* = 0.0001 for movement symmetry). On the other hand, the partial Sunnybrook scores based on symmetry at rest and on synkinesis were positively correlated with the SAQ score (Spearman’s rho: 0.4269, *p* = 0.0025 for Sunnybrook total; Spearman’s rho: 0.5165, *p* = 0.0002 for movement symmetry).

Regarding the functional outcomes related to eating and drinking, the logistic regression model found that increasing overall Sunnybrook scores were protective for the following conditions: slow chewing on the affected side (*p* = 0.004), lack of masticatory strength *p* = 0.025), masticatory fatigue (*p* < 0.001), accumulation of food in the oral vestibule (*p* < 0.001), difficulty in drinking from a glass (*p* = 0.019), and fluid spillage while drinking (*p* = 0.016). Moreover, increasing overall Sunnybrook scores were positively correlated with the probability of maintaining effective mastication on the affected side (*p* = 0.005). A detailed summary of the association between Sunnybrook scores (overall and partial) and each functional outcome (including odds ratios [ORs] with relative 95% confidence intervals [CI]) is reported in Table 3.

## 4. Discussion

The aim of this study was to investigate the impact of peripheral facial nerve paralysis after VS surgery on quality of life and analyze different factors which may contribute to the perceived quality of life in a sample of consecutive patients, with data retrieved in an outpatient postoperative rehabilitation setting, over a six-month period.

### 4.1. Overall Clinical Features

Most patients in our sample experienced paralysis only after surgery, since preoperative facial nerve function was normal in 46 out of 48 cases. Despite the surgical improvements over the years leading to a dramatic reduction in mortality and morbidity rates, transient and, rarely, definitive facial nerve palsy may still occur [7,8]. The issue of facial nerve preservation relates to surgical timing, patient selection, surgical approaches, and surgical dissection technique [25]. Successful preservation of the facial nerve is not always feasible, and iatrogenic damage has been reported [25].

Our sample’s patients showed different tumor sizes (median tumor size of 15 mm) and various degrees of postoperative facial palsy (Sunnybrook score: 68.0 [39.5–98.5]). According to the literature, in medium-to-large VS, a variable degree of postoperative facial nerve impairment is likely to result after surgery [3,4], potentially leading to permanent functional and aesthetic sequelae in up to half of the cases [9]. In spite of those findings, in the present study, a significant negative correlation was found between tumor size and the Sunnybrook score.

Even in the case of gross anatomical preservation, transient or definitive loss of facial nerve function may be caused by various possible traumatic mechanisms related to surgical dissection maneuvers [9]. Neurapraxia, the mildest degree of functional impairment (usually reversible), may be due to intraoperative nerve stretching, leading to an alteration in the axoplasmic membrane properties in the absence of any disruption of the axons and endoneurium [26,27]. Even in the absence of any gross anatomical lesion, more severe, long-term functional damages may be caused by nerve compression (possibly leading to axon and endoneurium disruption), by thermal damage (which can cause nerve coagulative necrosis), or by ischemic insults, due to the extensive manipulation during dissection maneuvers [28]. It is worth noting that the intracranial tract of the facial nerve is intrinsically more sensitive to mechanical stress than the extracranial part. In fact, proximally to the geniculate ganglion, the facial nerve shows a connective envelope made only of arachnoid, without the protection of epi- and perineurium sheaths. The perineurium and epineurium sheaths appear to be missing, since a clear fascicle pattern in axon distribution cannot be identified [29,30]. Such micro-anatomical features can reasonably explain both the higher sensitivity of the intracranial facial nerve to surgical traumas, and the higher likelihood of developing synkinesis during the reinnervation process, due to an intrinsic difficulty for the fibers to reach their physiological targets and the absence of strict fascicular organization [9,31].

### 4.2. Impact of Facial Nerve Damage in VS Surgery

As known from current clinical practice, postoperative facial nerve impairment remains one of the main functional concerns in VS surgery, despite the continuous refinement of surgical techniques and the subsequent overall reduction in morbidity [7,8,25]. The postoperative evaluation of facial nerve function is of utmost importance. Facial nerve objective dysfunctions and patients’ perception in everyday life are crucial, and postoperative clinical assessment involves both objective evaluations and patients’ reported outcomes of facial nerve function.

Several factors are involved in postoperative facial nerve function, including surgical timing, patient selection, surgical approaches, and dissection technique [25]. Early surgery usually allows for easier procedures, better chances of surgical radicality, more rapid recovery, and lower surgical morbidity (including better facial nerve outcomes in terms of functional preservation) compared with advanced cases [1,2,3,4,25]. In line with this view, the patients in our series showed a significant negative correlation between tumor size and the Sunnybrook score in terms of worse facial nerve outcomes in larger VS cases.

Also, further factors may affect the possibility of preserving facial nerve function in VS surgery. In particular, the intrinsic susceptibility of the intracranial tract of the facial nerve to physical trauma places it at risk of functional damage during surgery. 

In VS surgery, dissection maneuvers may cause a wide range of possible traumatic injuries, resulting in either temporary or permanent loss of function [9]. 

### 4.3. Clinical Relevance of Facial Nerve Function Parameters and Self-Reported Quality of Life Outcomes

Despite the development of multidimensional objective tools to evaluate facial nerve function, a comprehensive assessment of the clinical impact of facial nerve palsy on patients’ life remains a challenge [31,32].

When facial nerve palsy occurs in a preserved although damaged nerve, the timing to assess recovery is generally set between 12 and 18 months, although recovery takes place mostly in the first months after surgery. Conversely, when a graft is intraoperatively positioned because of facial nerve sacrifice, the timing of expected recovery is different and longer. A total of 9 to 12 months are necessary for the nerve to display any signs of recovery, although EMG at 6 months, in some cases, can show preliminary signs of regeneration [28,32,33]. In our sample, facial nerve reconstruction with graft was performed in 9 out of 48 patients. As shown in Table 1, the degree of facial palsy was significantly worse in those patients, but with no significant differences in terms of self-reported quality of life between patients who underwent facial nerve graft compared with those who did not. These data could support the hypothesis that each person reacts differently to facial nerve impairment, independently from the degree of surgical procedure. As reported by Moverare et al. [13], physicians should be aware that there is no positive correlation between the degree of facial palsy and the possible functional effect. Physicians are, therefore, recommended to ask specific questions relating to problems with these functions during customary medical visits and to offer a possible intervention by a speech–language therapist or a physiotherapist [13].

### 4.4. Sunnybrook Scores and Self-Reported Quality of Life 

In our investigation, the degree of facial palsy did not correlate with the perceived quality of life. Two domains were correlated to facial palsy severity: facial function (see Figure 2A–D) and general health (Figure 2E). Those data indicated a high level of accordance between the self-evaluation and the therapist’s evaluation of the functional impairment. We assume this was a positive effect of the early intervention that we currently perform in our clinic and as confirmed in other studies [34]. Early rehabilitation promotes faster development of awareness of the deficit and facial oral motor skills. In any rehabilitation phase of facial nerve deficiency, it is important that the first aim is the awareness of the patient and her/his knowledge of the face [35]. Patients need to control facial movements in a finer way than they did before surgery. For many authors, this is the basis of rehabilitation treatment, which is confirmed by our clinical experience [36,37].

### 4.5. Sunnybrook Scores and Self-Reported Facial Nerve Functional Outcomes

Patients who have a high perception of synkinesis tend to have worse facial palsy, with less symmetrical facial movements, more symmetry at rest, and higher presence of synkinesis. In our sample, as shown in Table 3, patients with a lower degree of facial palsy performed better while eating and drinking and tended to have fewer problems, such as slow chewing on the affected side, lack of masticatory strength, masticatory fatigue, accumulation of food in the oral vestibule, difficulty in drinking from a glass, and fluid spillage while drinking. Moreover, increasing overall Sunnybrook scores were positively correlated with the probability of maintaining effective mastication on the affected side. These data confirmed that facial nerve impairment impacts chewing on the injured side and thus determines less efficient bolus formation [14,15].

The assessment of outcomes in facial nerve palsy is an unsolved issue. Although some classifications, such as the House–Brackmann [17] and Sunnybrook Facial Grading System [18], allow for a viable way to measure (and compare) facial nerve function in preoperative and postoperative settings, some elements of recovery may not be tracked by those clinical tools. According to the results of the present study, it is important to also consider self-reported questionnaires in order to better understand the postoperative outcomes. Functional and aesthetic facial nerve impairments differently impact patients’ quality of life, independently of the schwannoma’s extent or surgical procedure. A complete preoperative and postoperative evaluation is warranted, and the role of the speech therapist is becoming increasingly important to take care of the patient in all her/his functional and aesthetic difficulties due to facial nerve palsy [11,37].

According to our in-house approach, the assessment has to be performed in the early postoperative days and be primarily focused on functional outcomes related to eating and drinking, as those are the first impaired functions that the patients experiment within the hospital setting. In the post-discharge weeks and months, once that eating and swallowing functions have been handled, facial asymmetry both at rest and during muscle contraction, as well as mimic non-verbal communication function [10,11], becomes the object of rehabilitation. In the case of facial graft, the role of rehabilitation is to ease the recovery of the nerve and prevent the establishing of synkinesis.

This study’s results should, however, be cautiously generalized, given its monocentric retrospective design and the relatively limited size of the considered series and its subgroups. However, the homogeneity of the included patients, who were all surgically treated by the same équipe, and the combined use of a multi-modal clinical facial nerve evaluation tool (namely, the Sunnybrook scale), along with patient-reported quality of life outcome measurements, represent some of the methodological strengths of this study.

## 5. Conclusions

Nowadays, facial nerve impairment is one of the most concerning issues in VS surgery, potentially impacting many aspects of patients’ life. Even with the limitation of this study design, this research study allowed the clinical impact of this condition on patients’ quality of life to be investigated, supporting the existence of an association between the degree of facial nerve damage (as defined by the overall and partial Sunnybrook scores) and the severity of the alteration in several functional aspects.

To better characterize the clinical impact of VS surgery-related facial nerve damage and the possible benefits from targeted rehabilitation strategies, further studies are advocated for, possibly with a multicenter prospective design.

## Figures and Tables

**Table 1:** Distribution of clinical and quality of life characteristics in the whole sample and across sub-groups.

Variable	Total (*N* = 48)				No Graft (*N* = 39)				Graft (*N* = 9)				*p*-Value *
	Median	IQR	Mean	SD	Median	IQR	Mean	SD	Median	IQR	Mean	SD	
Age (years)	52.5	45.5–59	52.1	11.8	52	46–59	52.4	12.6	53	44–59	51.0	8.2	0.5518
Tumor size (mm)	15	10–23	17.0	8.7	14	9–22	16.0	8.5	19.5	15–29	21.6	8.5	0.0794
Overall SBFGS ^§^	68	39.5–98.5	68.4	29.2	91	44–100	73.6	28.3	38	32–55	45.7	21.8	**0.0079**
SBFGS symmetry at rest ^§^	0	0–7.5	4.2	5.4	0	0–5	3.3	5.0	5	5–15	7.8	5.7	**0.0116**
SBFGS dynamic score ^§^	76	52–100	75.2	23.4	92	60–100	79.7	22.2	48	44–64	55.6	18.7	**0.0045**
SBFGS synkinesis ^§^	1.5	0–4.5	2.7	2.8	2	0–5	2.8	2.9	1	1–3	2.1	2.	0.7468
Overall PANQOL Scale	74.62	66.15–85.48	74.0	15.4	75.38	66.92–84.60	74.9	13.4	73.85	49.23–92.31	70.2	22.9	0.9473
PANQOL Anxiety	95	70–100	84.5	19.0	95	70–100	85.2	19.2	90	60–100	81.7	18.7	0.4038
PANQOL Facial Dysfunction	73.33	60.00–96.67	72.1	22.2	73.33	60.00–100.00	75.8	19.8	53.33	40.00–66.67	56.3	23.1	**0.0255**
PANQOL General Health	80	65–95	78.2	18.3	80	70–100	80.1	14.7	80	60–80	70.0	26.5	0.3893
PANQOL Balance	68.33	51.67–85.00	67.9	22.3	64.56	50.00–76.67	66.5	21.9	80.00	60.00–96.67	74.1	24.2	0.3076
PANQOL Hearing Loss	70	55–85	68.8	19.3	70	55–85	69.0	17.7	75	45–85	68.3	26.5	0.9051
PANQOL Energy	83.33	83.33–100.00	77.2	19.3	83.33	66.67–93.33	79.4	16.9	73.33	40.00–90.00	67.4	26.8	0.2131
PANQOL Pain	63.06	40.00–100-00	68.5	29.7	60.00	40.00–100.00	68.9	29.3	80.00	40.00–100.00	66.7	33.2	0.8373
SAQ score	22.22	17.78–34.44	28.4	13.3	22.22	17.78–35.56	28.3	13..5	24.44	20.00-31.11	28.9	13.1	0.4918

^§^ Postoperative SBFGS score. * Mann–Whitney U test. PANQOL Scale: Penn Acoustic Neuroma Quality of Life Scale; SAQ: Synkinesis Assessment Questionnaire, SBFGS: Sunnybrook facial grading system.

**Table 2:** Distribution of feeding outcomes in the whole sample and across subgroups. * Fisher’s exact test.

Functional Outcome		Total (*N* = 48)	No Graft (*N* = 39)		Graft (*N* = 9)		*p*-Value *
		*N* (%)	*N*	(%)	*N*	(%)	
Chewing on the affected side	No	12 (25.00)	9	(23.08)	3	(33.33)	0.671
	Yes	36 (75.00)	30	(76.92)	6	(66.67)	
Slow chewing	No	27 (56.25)	25	(64.10)	2	(22.22)	**0.031**
	Yes	21 (43.75)	14	(35.90)	7	(77.78)	
Lack of chewing strength	No	41 (85.42)	35	(89.74)	6	(66.67)	0.111
	Yes	7 (14.58)	4	(10.26)	3	(33.33)	
Chewing fatigue	No	33 (68.75)	31	(79.49)	2	(22.22)	**0.002**
	Yes	25 (31.25)	8	(20.51)	7	(77.78)	
Effective lip seal	No	12 (25.00)	9	(23.08)	3	(33.33)	0.671
	Yes	36 (75.00)	30	(76.92)	6	(66.67)	
Food accumulation in the oral vestibule	No	21 (43.75)	20	(51.28)	1	(11.11)	0.058
	Yes	27 (56.25)	19	(48.72)	8	(88.89)	
Cheek biting	No	30 (62.50)	27	(79.23)	3	(33.33)	0.063
	Yes	18 (37.50)	12	(30.77)	6	(66.67)	
Difficulty in drinking from a glass	No	41 (85.42)	34	(87.18)	7	(77.78)	0.601
	Yes	7 (14.58)	5	(12.82)	2	(22.22)	
Liquid spillage while drinking	No	34 (70.83)	26	(66.67)	8	(88.89)	0.250
	Yes	14 (29.17)	13	(33.33)	1	(11.11)	
Difficulty in retaining saliva	No	41 (85.42)	33	(84.62)	8	(88.89)	1.000
	Yes	7 (14.58)	6	(15.38)	1	(11.11)	
Other feeding impairment	No	46 (95.83)	38	(97.44)	8	(88.89)	0.343
	Yes	2 (4.17)	1	(2.56)	1	(11.11)	

**Table 3:** Logistic regression model showing the association between Sunnybrook scores and feeding outcomes.

Functional Outcome	Overall SBFGS Score		Symmetry at Rest Score		Movement Symmetry Score		Synkinesis Score	
	OR (95% C.I.)	*p*-Value	OR (95% C.I.)	*p*-Value	OR (95% C.I.)	*p*-Value	OR (95% C.I.)	*p*-Value
Chewing on the affected side	1.042 (1.013–1.073)	**0.005**	0.808 (0.704–0.927)	**0.002**	1.046 (1.012–1.082)	**0.009**	0.794 (0.628–1.002)	0.052
Slow chewing	0.968 (0.946–0.990)	**0.004**	1.190 (1.046–1.354)	**0.008**	0.961 (0.935–0.989)	**0.006**	1.154 (0.935–1.426)	0.182
Lack of chewing strength	0.956 (0.918–0.994)	**0.025**	1.247 (1.062–1.463)	**0.007**	0.950 (0.907–0.993)	**0.027**	1.050 (0.795–1.385)	0.733
Chewing fatigue	0.931 (0.894–0.969)	**<0.001**	1.289 (1.112–1.495)	**0.001**	0.914 (0.870–0.961)	**<0.001**	1.200 (0.964–1.494)	0.103
Effective lip seal	1.002 (0.980–1.025)	0.867	0.963 (0.856–1.084)	0.534	1.003 (0.975–1.031)	0.857	1.135 (0.873–1.477)	0.345
Food accumulation in the oral vestibule	0.944 (0.916–0.973)	**<0.001**	1.265 (1.069–1.497)	**0.006**	0.926 (0.890–0.964)	**<0.001**	1.123 (0.975–1.548)	0.086
Cheek biting	0.981 (0.961–1.002)	0.081	1.135 (1.011–1.274)	**0.033**	0.977 (0.952–1.003)	0.082	0.932 (0.750–1.158)	0.523
Difficulty in drinking from a glass	0.946 (0.901–0.991)	**0.019**	1.247 (1.062–1.463)	**0.007**	0.941 (0.894–0.990)	**0.019**	1.176 (0.901–1.537)	0.233
Liquid spillage while drinking	0.971 (0.948–0.995)	**0.016**	1.154 (1.024–1.301)	**0.019**	0.968 (0.940–0.997)	**0.030**	1.360 (1.069–1.731)	**0.012**
Difficulty in retaining saliva	0.977 (0.949–1.007)	0.136	1.144 (0.993–1.317)	0.062	0.976 (0.941–1.012)	0.180	1.112 (0.850–1.456)	0.439
Other feeding impairment	0.961 (0.898–1.029)	0.254	1.109 (0.876–1.404)	0.389	0.954 (0.880–1.034)	0.248	1.232 (0.786–1.903)	0.363

SBFGS: Sunnybrook facial grading system.

## Data Availability

The data presented in this study are available upon request from the corresponding author.
